# Capacity, Collaboration, and Thematic Structure in Health AI Research Over the Past Decade in the Gulf Cooperation Council: Scientometric Analysis

**DOI:** 10.2196/105709

**Published:** 2026-10-21

**Authors:** Ahmed Alqheedan, Saleh Alzughaibi

**Affiliations:** 1 Department of Health Informatics, College of Health Sciences, Saudi Electronic University, Abi Bakr As Siddiq Rd, Ar Rabi, Riyadh, Riyadh Region, 13316, Saudi Arabia, 966 115000333

**Keywords:** artificial intelligence, AI, bibliometrics, scientometrics, digital health, Gulf Cooperation Council, research capacity, OpenAlex, health informatics

## Abstract

**Background:**

Health AI investment is accelerating across the Gulf Cooperation Council (GCC), yet the region’s health AI research output has never been systematically quantified. National research capacity is a structural precondition for the safe local development and governance of clinical AI, but no scientometric baseline exists for the 6 GCC states.

**Objective:**

We characterized the volume, growth, collaboration structure, and thematic composition of GCC health AI research and examined whether national output aligns with an existing measure of informatics readiness.

**Methods:**

We conducted an ecological scientometric analysis of all health AI publications affiliated with the 6 GCC states (Bahrain, Kuwait, Oman, Qatar, Saudi Arabia, and the United Arab Emirates) for 2011 to 2024 (the last complete indexing year at extraction) using the open bibliographic database OpenAlex. A publication was eligible if it included both an AI family and a health or medicine family concept above a 0.30 relevance threshold. Bibliometric indicators (output, compound annual growth, per capita intensity, citation impact, international and intraregional collaboration, and institutional concentration) were computed; coauthorship and concept co-occurrence networks were constructed and clustered by modularity. National health AI intensity was correlated (Spearman) with the Health Informatics Readiness Index (HIRI) and Global Digital Health Monitor (GDHM) phase.

**Results:**

Of 112,480 GCC-affiliated AI publications, 3512 met the health AI definition. Regional output rose from 18 (2011) to 904 (2024) publications per year (compound annual growth=35.2%), with 86.4% (3034/3512) published from 2020 onward and a sharp inflection from 2019 to 2021. Saudi Arabia contributed 59.6% (2094/3512) of the output by itself and 81.5% (2862/3512) together with the United Arab Emirates; Qatar led on a per capita basis (approximately 136 publications per million population). International collaboration (2551/3512, 72.6% of the papers) predominated over intra-GCC collaboration (164/3327, 4.9%), with the latter being inversely related to national output. The principal external partners were India, the United States, Pakistan, the United Kingdom, and Egypt. Output spanned 3186 institutions; concentration ranged from 89.4% (328/367) of a country’s output among the top 3 institutions for Qatar to 34.4% (721/2094) for Saudi Arabia. Concept co-occurrence was classified into 4 themes—classic machine learning clinical prediction, medical imaging and oncology, COVID-19 response, and diabetic eye disease. Health AI intensity (ρ=+0.60) and, more strongly, absolute output (ρ=+0.89) correlated with the HIRI; absolute output was also correlated with GDHM phase (ρ=+0.83).

**Conclusions:**

GCC health AI research has grown rapidly but unevenly, is heavily dependent on international collaboration, is concentrated in medical imaging, and—outside Saudi Arabia—remains anchored in single national institutions. Informatics readiness was associated with both the intensity and the volume of national research output. The findings establish a reproducible baseline for monitoring regional health AI research capacity as GCC health systems move toward clinical AI deployment.

## Introduction

AI is moving from research prototypes into the operational core of health systems, and national investment is accelerating worldwide under frameworks such as the World Health Organization’s (WHO) *Global Strategy on Digital Health 2020-2025* [1]. The capacity of a country to produce, absorb, and govern health AI knowledge is increasingly treated as a structural determinant of whether clinical AI can be deployed safely and equitably. A recent ecological panel study across the 27 European Union member states found that national health AI research capacity did not translate automatically into population-level patient value and that the association was strongly moderated by financial protection context and the COVID-19 period [2]. This work demonstrates both the analytic value of treating health AI research capacity as a measurable national attribute and the limits of assuming that capacity alone improves outcomes.

No equivalent baseline exists for the Gulf Cooperation Council (GCC). The 6 GCC states—Bahrain, Kuwait, Oman, Qatar, Saudi Arabia, and the United Arab Emirates (UAE)—simultaneously are among the world’s most active digital health investors and, although Gulf health and eHealth research has been surveyed bibliometrically at a broader level [3-5], have not been characterized specifically in terms of health AI research capacity using a validated, reproducible measurement approach [6,7]. Existing instruments capture adjacent but distinct constructs: the Global Digital Health Monitor (GDHM) measures digital health maturity [6], the universal health coverage service coverage index measures service outputs [8], and the International Telecommunication Union (ITU) ICT Development Index measures connectivity [9]. None quantifies the research base from which locally relevant clinical AI is developed.

Scientometric analysis offers a direct, reproducible route to that measurement. Bibliometric methods are now well codified for both performance analysis and science mapping [10-12], and the emergence of fully open bibliographic infrastructure—notably OpenAlex—removes the cost and access barriers that previously limited such work in lower-resourced settings [13]. We applied these methods to the GCC to establish the first regional baseline of health AI research capacity and test whether that capacity aligned with an independent measure of informatics readiness for the same 6 countries [14].

This study addresses four questions:

1. What is the volume, growth, and per capita intensity of health AI research output across the 6 GCC states (2011‐2024)?
2. What is the collaboration structure—international vs intraregional—and which institutions anchor it?
3. What thematic clusters characterize GCC health AI research, and how have they evolved?
4. Does national output align with informatics readiness (Health Informatics Readiness Index; HIRI) and digital health maturity (GDHM phase)?

## Methods

### Study Design and Reporting

This was an ecological, country-level scientometric analysis of the 6 GCC states for the period from 2011 to 2024. Record selection is reported following the PRISMA (Preferred Reporting Items for Systematic Reviews and Meta-Analyses) 2020 guidelines [15], and observational reporting follows the STROBE (Strengthening the Reporting of Observational Studies in Epidemiology) statement adapted to aggregate bibliographic data [16]. The unit of analysis was the publication; aggregation was to country and country-year. Publications were attributed to each GCC country represented among their affiliations (full counting), so regional totals count a publication once for each GCC country involved; the 3512 country-attributed records, and the record-selection counts in Figure 1, therefore correspond to 3327 distinct publications. Regional intra-GCC collaboration is reported as a share of distinct publications, because full counting would count each such publication at least twice.

### Data Source and Eligibility

Bibliographic records were obtained from OpenAlex, a fully open index of scholarly works with institution, country, concept, and citation metadata [17]. OpenAlex was selected over subscription databases for its open access, programmatic reproducibility, and comparable coverage. We note that OpenAlex concepts (derived from the Microsoft Academic Graph) are no longer actively maintained and have been superseded by OpenAlex topics; because the frozen AI family vocabulary predates the large language model era, works indexed only under newer terms such as “large language model,” “transformer,” or “generative AI” may be undercounted, a limitation we quantify and discuss below. Records were retrieved via the public API on the extraction date (June 2026), filtered to works affiliated with a GCC institution, tagged with the OpenAlex concept artificial intelligence [18], and with a publication year of 2011 to 2024. Records dated as 2025 were present at extraction but were excluded because 2025 was an incomplete, still accruing year subject to indexing lag at the retrieval date (June 2026); restricting the corpus to 2011 to 2024 ensured that every analyzed year was a complete indexing year and that growth estimates were not distorted by a partially indexed final year. To assess robustness to database choice, the same population was independently retrieved from Scopus using a title-abstract-keyword query combining AI and health terms with GCC affiliation and year filters, and the 2 sources were compared on annual output, national distribution, and record-level overlap.

A work was defined as *health AI* if it included at least one AI family concept (AI, machine learning, deep learning, or artificial neural networks) *and* at least one health or medicine family concept, each with a concept relevance score of 0.30 or higher. The health or medicine family comprised 6 OpenAlex concepts: medicine, internal medicine, intensive care medicine, family medicine, health care, and public health. The complete concept identifiers and relevance scores for both families are provided in the deposited extraction script [19]. Both concept lists and the threshold were prespecified and are reported as sensitivity parameters. Each work in OpenAlex includes, for every assigned concept, a classifier-generated relevance score in the range from 0 to 1 that reflects the confidence of the automated concept-tagging model [20]; low scores predominantly correspond to weak, incidental assignments. We adopted a 0.30 cutoff to exclude such marginal tags while retaining substantive ones and tested its influence directly: in a sensitivity check, raising the threshold to 0.40 or lowering it to 0.20 changed absolute counts but left the national ranking, growth trajectory, and thematic structure unchanged. To estimate precision, 2 authors manually adjudicated a random sample of 100 included records against title and topic; 94 were unambiguous health AI applications (95% Wilson CI 87.5%‐97.2%), and no clearly off-topic record was identified, with the remainder comprising borderline health informatics works rather than misclassifications. This definition prioritizes specificity over recall and is fully reproducible from the deposited extraction script. Throughout, “health AI” denotes this concept-defined corpus; “clinical AI” is used only for the narrower subset of AI intended for point-of-care clinical use.

### Indicators and Analysis

For each country, we computed total and annual output, compound annual growth rate (CAGR), output per million population (World Bank midyear estimates) [21], mean and median citations, the proportion of works with at least one non-GCC affiliation (international collaboration) and with 2 or more GCC state affiliations (intraregional collaboration), and the share of national output accounted for by the 3 most prolific institutions. Coauthorship was analyzed at the country level; thematic structure was analyzed by constructing a concept co-occurrence network and partitioning it into clusters through modularity maximization [22]. Thematic evolution was assessed by classifying each work’s primary topic into macrothemes across the period.

To address question 4, national health AI output and per capita intensity were correlated with the equal-weight HIRI [14] and with GDHM maturity phase [6] using Spearman rank-order coefficients. The HIRI is an equal-weight composite index of national health informatics readiness for the 6 GCC states, constructed from ITU, WHO Global Health Observatory, GDHM, and World Bank indicators spanning digital infrastructure, workforce and governance, and financing [14]. The GDHM is a global instrument that classifies countries into ordinal maturity phases across governance, infrastructure, services, and data domains of digital health development [6]. With 6 countries, statistical power was minimal; coefficients are reported descriptively by magnitude and direction rather than against significance thresholds. Analyses used Python (version 3.11; Python Software Foundation; Pandas, NetworkX, and Matplotlib libraries); all data and code are reproducible from the tabulated inputs.

### Ethical Considerations

This study did not involve human participants, human tissue, or individual-level patient data; it used only aggregate, publicly available bibliographic metadata retrieved from OpenAlex together with publicly reported country-level indicators. Under the applicable national research governance framework and the policies of Saudi Electronic University, secondary analyses of publicly available, nonidentifiable aggregate data of this kind do not require review by an institutional review board or research ethics committee, and no such application was therefore submitted. No informed consent was required as no human participants were involved.

## Results

### Record Selection

Of 112,480 AI publications affiliated with GCC institutions over 2011 to 2024, a total of 108,968 were excluded for including no health or medicine concepts at the 0.30 threshold, leaving 3512 (3.1%) health AI publications for analysis among all the GCC AI research output throughout the period (Figure 1).

**Figure 1.**
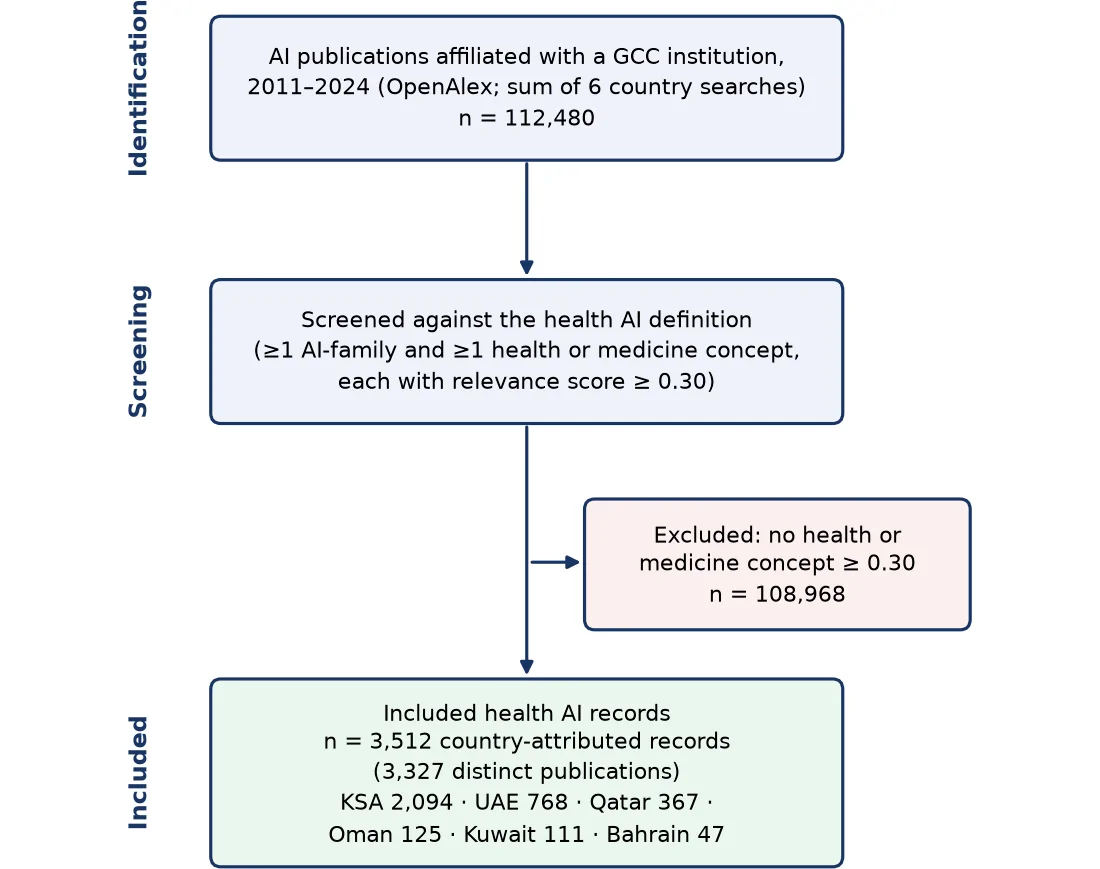
Record selection (PRISMA [Preferred Reporting Items for Systematic Reviews and Meta-Analyses]-style flow diagram). Of 112,480 AI publications affiliated with Gulf Cooperation Council (GCC) institutions over 2011 to 2024, a total of 108,968 were excluded for including no health or medicine concepts at the 0.30 relevance threshold, leaving 3512 health AI publications for analysis. KSA: Kingdom of Saudi Arabia; UAE: United Arab Emirates.

### Database Concordance

The independent Scopus query retrieved 14,676 records for 2011 to 2024 (15,560 country attributions, counted in the same way as for OpenAlex). The broader keyword strategy returned a larger corpus than the concept-based OpenAlex definition (n=3512), but the 2 databases were highly concordant on aggregate structure (Figure 2). Annual output was almost perfectly correlated (Spearman ρ=0.99; Pearson *r*=0.99), with both sources showing the same 2019 to 2021 inflection. The national distribution was identical in rank order (Spearman ρ=1.00): Saudi Arabia was first in both (2094/3512, 59.6% of the OpenAlex output and 9463/15,560, 60.8% of the Scopus output), followed by the UAE (768/3512, 21.9% of the OpenAlex output and 3376/15,560, 21.7% of the Scopus output), Qatar, Oman, Kuwait, and Bahrain, with national shares differing by no more than 1.8 percentage points between sources. At the record level, 58.6% (1951/3327) of distinct OpenAlex titles had an exact normalized title match in the larger Scopus set, a lower bound given that title normalization does not capture near matches. The substantive findings reported below are therefore robust to database choice.

Regional health AI output rose from 18 publications in 2011 to 904 in 2024, a CAGR of 35.2%, with 86.4% (3034/3512)of the corpus published from 2020 onward. Output inflected sharply from 2019 to 2021, when annual production more than trebled from 135 to 435 publications (Figure 3).

**Figure 2.**
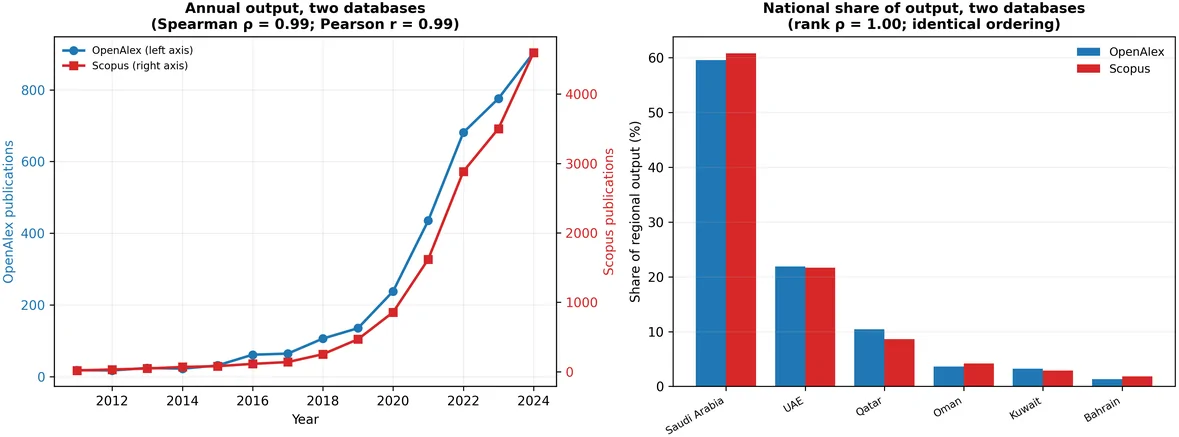
Database concordance between OpenAlex and Scopus: (left) annual health AI output (2011‐2024; Spearman ρ=0.99) and (right) national share of regional output by database; rank order was identical (ρ=1.00), and shares differed by ≤1.8 percentage points. Axes for the 2 databases are scaled independently in panel A because the broader Scopus keyword query returned a larger corpus (n=14,676) than the concept-based OpenAlex definition (n=3512). UAE: United Arab Emirates.

**Figure 3.**
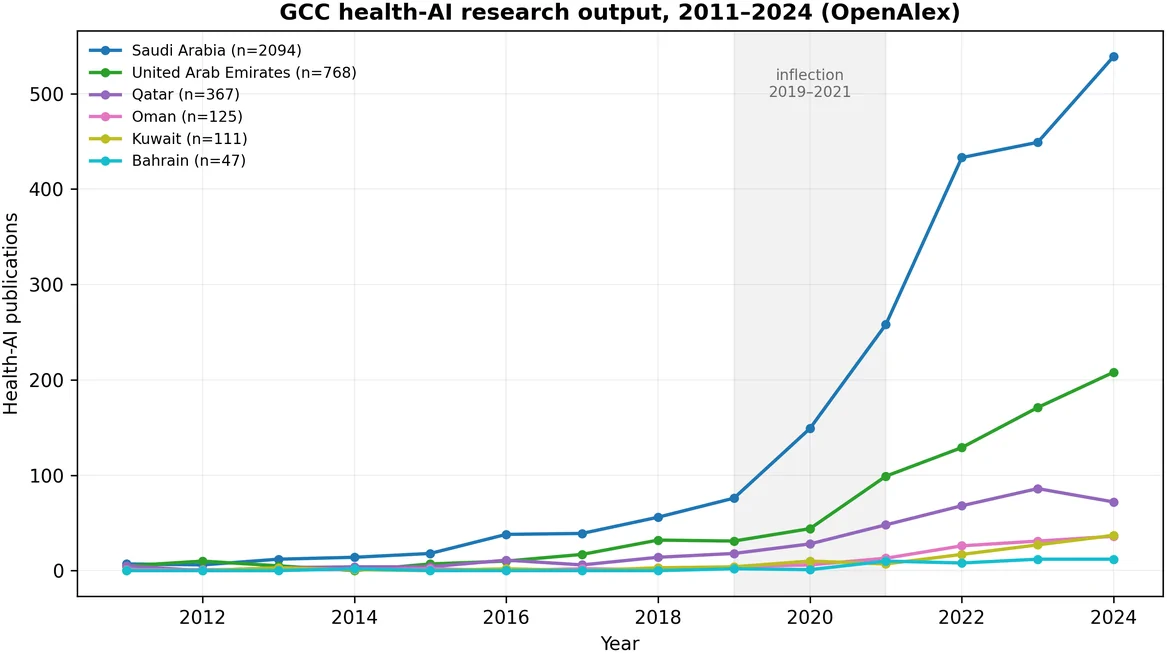
Annual health AI publications by Gulf Cooperation Council state (2011‐2024). All 6 trajectories increased; regional output inflected from 2019 to 2021.

### National Distribution and Research Intensity

Output was strongly concentrated: Saudi Arabia produced 59.6% (2094/3512) of the corpus by itself and 81.5% (2862/3512) together with the UAE (Table 1). Normalized to population, the ranking was reordered, with Qatar leading at approximately 136 publications per million population ahead of the UAE (n=81) and Saudi Arabia (n=63)—indicating that absolute volume reflects national scale rather than research intensity.

**Table 1.** Health AI research indicators for the 6 Gulf Cooperation Council (GCC) states (2011‐2024; n=3512 country-attributed records; n=3327 distinct publications)^a^.

Country	Works, n (%)	Works per million population^b^, n	International collaboration, n (%)	Intra-GCC collaboration, n (%)	Works from the top 3 institutions, n^c^ (%)	Citations^d^, mean (SD)	Citations^d^, mean (IQR)
Saudi Arabia	2094 (59.6)	62.9	1512 (72.2)	133 (6.4)	721 (34.4)	29.6 (85.2)	11 (3-32)
United Arab Emirates	768 (21.9)	80.8	575 (74.9)	106 (13.8)	295 (38.4)	25.6 (60.0)	8 (2-27.25)
Qatar	367 (10.4)	135.9	258 (70.3)	51 (13.9)	328 (89.4)	42.3 (182.4)	12 (2-35)
Oman	125 (3.6)	27.2	95 (76.0)	19 (15.2)	70 (56.0)	14.2 (38.3)	5 (1-14)
Kuwait	111 (3.2)	25.8	84 (75.7)	24 (21.6)	61 (55.0)	19.1 (27.3)	7 (1-26.5)
Bahrain	47 (1.3)	31.3	27 (57.4)	16 (34.0)	22 (46.8)	19.3 (33.2)	8 (3-17)

a Full counting was used, so a publication coauthored by institutions in 2 or more GCC states is counted for each state. Country rows therefore sum to 3512 records from 3327 distinct publications, and the intra-GCC counts sum to 349 attributions from 164 distinct publications (4.9%), the figure reported in the text.

b Populations are approximate 2024 World Bank estimates.

c Summed publication counts of each country’s 3 most prolific institutions as a share of national output; a paper coauthored by 2 of these institutions is counted for each.

d OpenAlex citation counts at data extraction. Distributions are highly right-skewed: the most cited record (3287 citations; Qatar) inflates Qatar’s mean and SD. Means are also confounded by article age and should be read alongside the medians. SD: sample SD. IQR: 25th-75th percentile.

### Collaboration Structure

International collaboration predominated throughout: 72.6% (2551/3512)of the corpus had at least one non-GCC affiliation compared to 4.9% (164/3327) of distinct publications coauthored *across 2 or more GCC states*. Internationalization rose across the 3 subperiods (70/112, 62.5% in 2011‐2015; 411/604, 68% in 2016‐2020; and 2070/2796, 74% in 2021‐2024), as did intra-GCC collaboration among distinct publications (0/112, 0% in 2011-2015; 6/596, 1% in 2016-2020; and 158/2619, 6% in 2021-2024), indicating regional networks that formed late and remain modest. Intra-GCC collaboration was inversely related to national output, reaching 34% (16/47) in Bahrain and 21.6% (16/47) in Kuwait but only 6.4% (133/2094) in Saudi Arabia, consistent with smaller research systems relying on regional partners while the largest were comparatively self-sufficient. Across 113 partner countries, the principal external collaborators were India (n=598 publications), the United States (n=556), Pakistan (n=410), the United Kingdom (n=393), and Egypt (n=297; Figure 4) [23].

**Figure 4.**
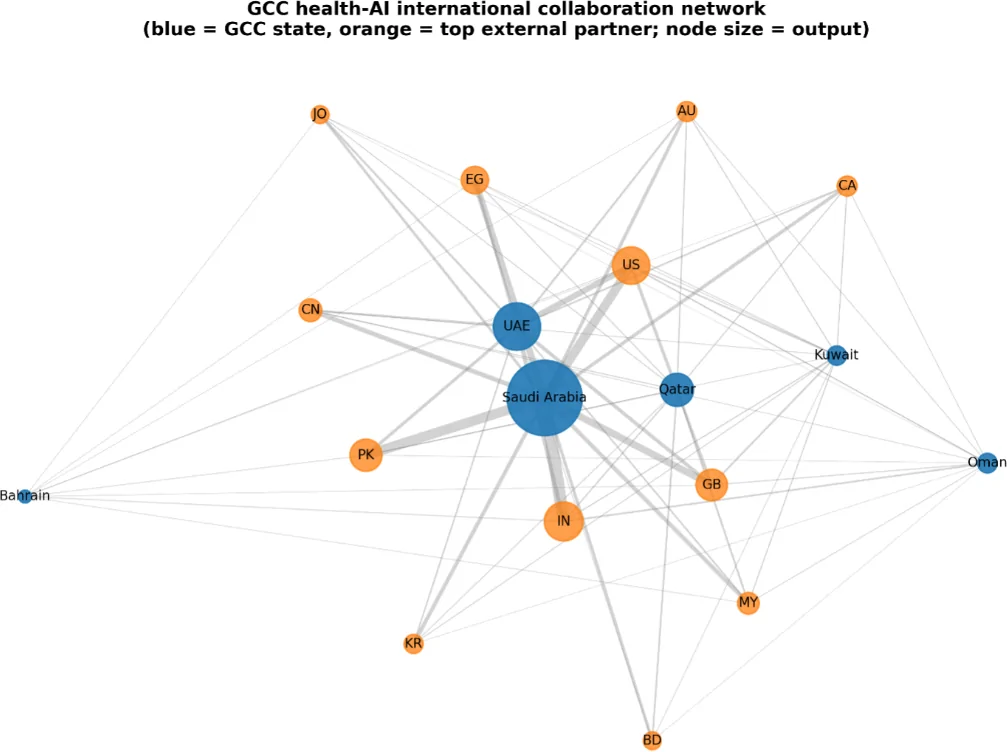
International collaboration network. Blue nodes are Gulf Cooperation Council states; orange nodes are the 12 most frequent external partner countries. Node size is proportional to output, and edge width is proportional to the number of coauthored publications. Data source: OpenAlex. AU: Australia; BD: Bangladesh; CA: Canada; CN: China; EG: Egypt; GB: United Kingdom; IN: India; JO: Jordan; KR: South Korea; MY: Malaysia; PK: Pakistan; UAE: United Arab Emirates; US: United States.

### Institutional Concentration

Output was distributed across 3186 distinct institutions. The most prolific were King Saud University (n=328 publications), King Abdulaziz University (n=249), Princess Nourah bint Abdulrahman University (n=195), and Qatar University (n=175). Concentration varied markedly: the 3 leading institutions accounted for 89.4% (328/367)of Qatar’s output but only 34.4% (721/2094)of Saudi Arabia’s and 38.4% (295/768) of the UAE’s (Table 1), distinguishing nationally distributed research systems from those anchored in a single flagship university.

### Thematic Structure and Evolution

Concept co-occurrence was classified into 4 coherent clusters (Figure 5), each labeled here by its most frequent constituent concepts (in parentheses): classic machine learning clinical prediction (random forest, support vector machines, logistic regression, and internal medicine), medical imaging and oncology (deep learning, transfer learning, segmentation, breast cancer, magnetic resonance imaging, pathology, and radiology), COVID-19 response (SARS-CoV-2, intensive care, and pandemic), and diabetic eye disease (diabetic retinopathy, ophthalmology, and optometry). Temporally, AI-based COVID-19 research surged from 2020 to 2022 before receding as a share, whereas research on oncology and imaging and on general AI in health remained the persistent leaders (Figure 6). The single most cited work in the corpus was a 2017 deep learning study of lymph node metastasis detection with Qatari coaffiliation (3287 citations) [24].

**Figure 5.**
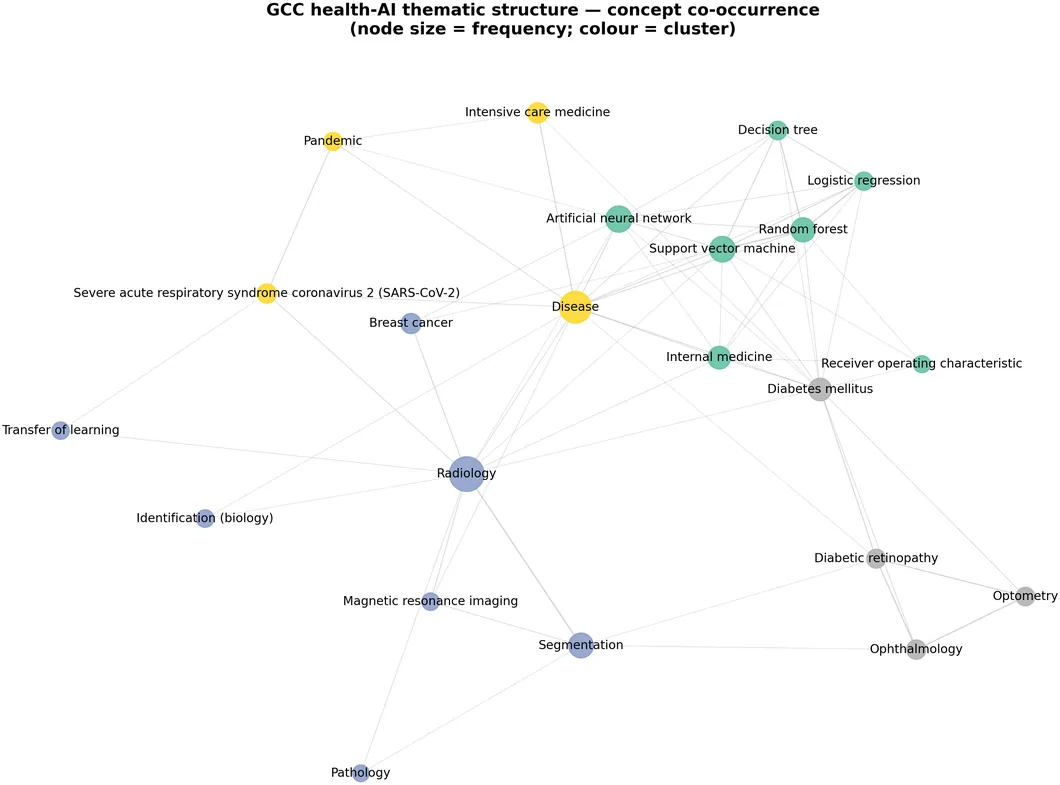
Thematic structure of Gulf Cooperation Council health AI research by concept co-occurrence. Node size denotes concept frequency; color denotes modularity cluster. Four themes emerged: classic machine learning clinical prediction, medical imaging and oncology, COVID-19 response, and diabetic eye disease.

**Figure 6.**
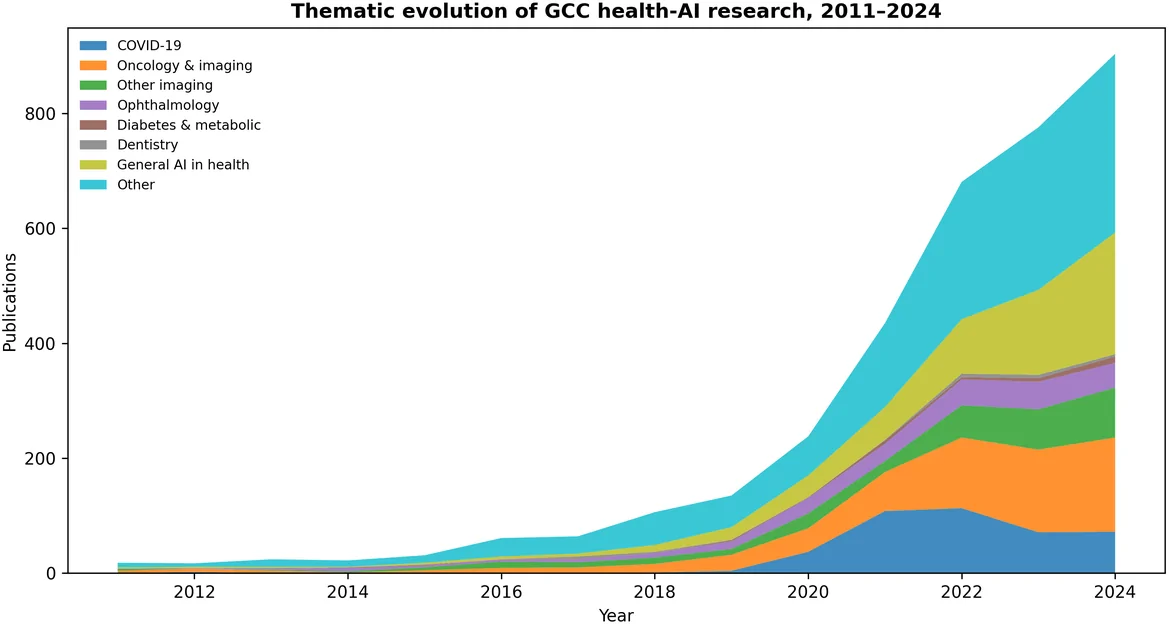
Thematic evolution (2011‐2024) by macrotheme. AI-based COVID-19 research surged from 2020 to 2022; the persistent leaders were research on oncology and imaging and on general AI in health. The macrotheme classification is approximate and is provided for descriptive purposes; the concept co-occurrence network is the primary thematic representation (see the Limitations section).

### Association With Informatics Readiness

National health AI research intensity (publications per million population) correlated positively with the HIRI (Spearman ρ=+0.60), and absolute output correlated more strongly (ρ=+0.89). Absolute output was also correlated with GDHM maturity phase (ρ=+0.83; Figure 7). In a sensitivity analysis recomputing the correlations across the 19 alternative index specifications defined in the HIRI preprint [14,25], the correlation with absolute output ranged from +0.66 to +0.94 and that with intensity from +0.31 to +0.66 (Multimedia Appendix 1), so readiness was associated more consistently with the volume of national research than with its per capita intensity. Because this analysis was based on only 6 observations, we treat it as exploratory rather than confirmatory; the per-country HIRI and GDHM values are reported in Multimedia Appendix 1. The pattern is consistent with the global association between digital maturity and health system performance [25].

**Figure 7.**
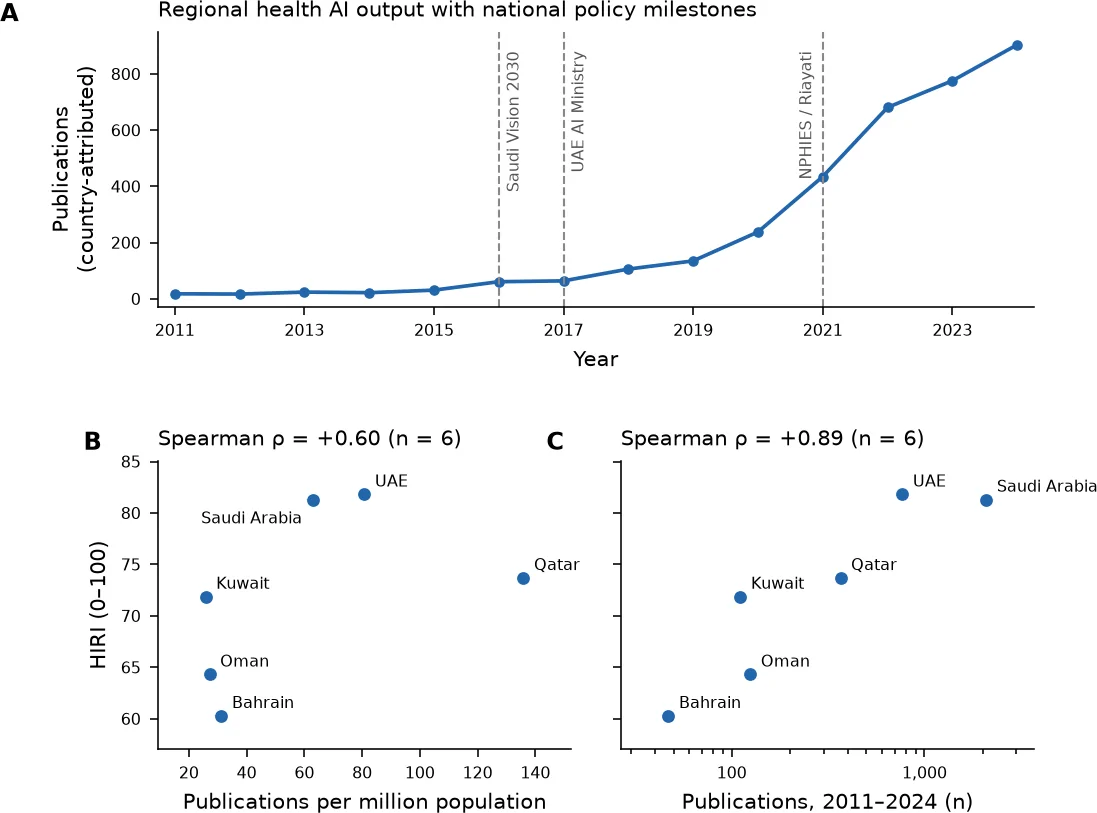
(A) Regional output annotated with national policy milestones and (B) national health AI intensity (publications per million population) and (C) absolute health AI output, each vs the Health Informatics Readiness Index (HIRI; Spearman ρ=+0.60 and ρ=+0.89, respectively; n=6 countries). NPHIES: National Platform for Health and Insurance Exchange Services; UAE: United Arab Emirates.

## Discussion

### Principal Findings

This is the first scientometric baseline of health AI research for the GCC. Earlier bibliometric studies have mapped adjacent slices of Gulf research: Weber et al [3] reviewed eHealth research across the same 6 states; Al-Farsi et al [4] standardized Gulf health research output to population and identified Qatar as the leading country on that basis; and Al-Marzouqi and Arabi [5] normalized regional output to population, gross domestic product, research and development expenditure, and researcher counts, reporting Saudi dominance in absolute volume, Qatar as having the steepest relative growth, and high international collaboration. Our per capita and collaboration findings are consistent with this previous work; the specific contribution here is the restriction of the lens to health AI; the addition of a concept-level thematic structure and an explicit readiness linkage; and, thereby, the characterization of the research base from which locally relevant clinical AI must be developed rather than of Gulf health research as a whole. Four findings stand out. First, output grew rapidly (CAGR=35.2%) but from a low base and with a pronounced post-2019 inflection that coincided with the diffusion of deep learning and with national digital health milestones. Second, the research base is heavily internationalized: nearly three-quarters of the publications involved a non-GCC partner, and the proportion of intraregional collaboration, though rising, remained under 1 in 10 papers. Third, the field was concentrated on medical imaging and, outside Saudi Arabia and the UAE, anchored in a single national institution. Fourth, informatics readiness as captured by the HIRI was associated with both research intensity and absolute output, most consistently with output, as was digital health maturity.

The contrast with the aforementioned European Union panel study is instructive [2]. Where that study found that health AI research capacity did not automatically improve population-level value, the present analysis was deliberately upstream: it measured capacity itself before any outcome linkage. The strong external partner structure—dominated by India, the United States, Pakistan, the United Kingdom, and Egypt—reflects both the GCC’s reliance on an internationally recruited research workforce and the regional and diaspora ties that shape Gulf science [23]. This dependency is invisible to connectivity or maturity indexes and is a distinctive contribution of the scientometric lens. We report indicators in absolute and per capita terms without a formal world, Middle East and North Africa, or matched country baseline; nonetheless, the international collaboration share (2551/3512, 72.6%) was high relative to the roughly one-fifth to one-quarter international coauthorship reported worldwide, and the 35.2% compound annual growth exceeds the global growth of AI publishing over the same period, indicating that the GCC pattern is not merely a reflection of worldwide trends. The absence of a matched external comparator is a limitation and a priority for future work.

The positive association of the HIRI with both research intensity (ρ=+0.60) and output (ρ=+0.89) suggests that informatics readiness and research capacity develop together, although with 6 countries this remains exploratory. The concentration of output on imaging mirrors the global health AI literature and signals an opportunity: domains underrepresented in the GCC corpus—primary care, mental health, health systems, and safety research—are precisely those where locally generated evidence is most needed before deployment.

### Policy Implications

#### Overview

The findings carry concrete implications for the region’s national digital health agendas. The post-2019 inflection in output coincides with the operationalization of Saudi Arabia’s Health Sector Transformation Program under Vision 2030 [26], the UAE’s AI governance institutions, and Qatar’s National Vision 2030 health information programs, indicating that research capacity has responded to, and can be steered by, deliberate national policy. We frame the country-specific recommendations below as policy hypotheses rather than as conclusions that the bibliometric evidence establishes on its own: our data measure publication counts, national shares, and coauthorship patterns, not efficiency, institutional resilience, or the returns to coordinated investment, and the prescriptions should be read as directions warranting evaluation rather than demonstrated causal claims.

#### Saudi Arabia

As the source of 3 in 5 regional publications but only the third-ranked country on research intensity, Saudi Arabia generates volume commensurate with both its scale and its informatics readiness, on which it ranks second in the region. Its comparatively distributed institutional base (34.4% of its output was from the top 3 institutions) is a national asset that Vision 2030’s research and development targets and the National Health Information Center should consolidate—by directing health informatics workforce investment toward the governance and applied safety domains that remain underrepresented, in line with documented competency gaps among Saudi health informatics graduates [27,28].

#### UAE and Qatar

Both countries lead on research intensity but depend on a narrow institutional base—acutely so in the case of Qatar, where the top 3 institutions accounted for 89.4% of its output. Their national AI in health strategies should prioritize broadening the productive base beyond flagship universities and academic medical centers so that capacity is resilient to the departure of individual research groups and the internationally recruited workforce on which it currently rests.

#### Bahrain, Kuwait, and Oman

These smaller systems already collaborate the most within the region (intra-GCC collaboration: 16/47, 34% in Bahrain and 24/111, 21.6% in Kuwait), positioning them to benefit disproportionately from shared regional infrastructure. For these states, the efficient route to capacity is pooled rather than purely national investment.

#### Regional and WHO Eastern Mediterranean Region

Two structural gaps cut across all 6 states and align directly with the WHO Eastern Mediterranean Region digital health strategy for 2023 to 2027: low intraregional collaboration (under 1 paper in 10) and thematic narrowness concentrated on medical imaging at the expense of primary care, mental health, and health systems and patient safety research. A coordinated GCC health AI research program—shared datasets, joint funding calls, and a common evaluation framework—would address both and ensure that locally generated evidence from underserved clinical domains is in place before, not after, clinical AI is deployed at scale [29].

### Limitations

Four limitations should be considered. The health AI definition is concept based and trades recall for precision; manual audit indicated high precision (94%), but recall was not estimated, and alternative thresholds shift absolute counts but not the comparative structure. In addition, because eligibility relied on the now deprecated OpenAlex concept vocabulary, the corpus underrepresents very recent large language model, transformer, and generative AI work indexed chiefly under newer terms; this most plausibly affects the 2022 to 2024 segment and means that the headline growth curve, if anything, understates the most recent expansion. The primary corpus was derived from OpenAlex; although a Scopus concordance check confirmed that the temporal and geographic structure was robust to database choice (Figure 2), affiliation disambiguation was imperfect, and English-language indexing undercounted Arabic-language output in both sources. The macrotheme classification used for thematic evolution was coarse, leaving a substantial unclassified residual; the concept co-occurrence network is the more rigorous thematic representation. Finally, the 6-country correlation with the HIRI and GDHM is descriptive and cannot support inferential or causal claims.

### Conclusions

GCC health AI research has expanded rapidly but unevenly, depends heavily on international collaboration, is concentrated on medical imaging, and—outside Saudi Arabia—remains institutionally narrow. Informatics readiness was associated with both the intensity and the volume of national research output. These results provide a reproducible baseline against which the region’s health AI research capacity can be monitored as GCC health systems move toward the deployment of clinical AI, and they identify regional collaboration and thematic breadth as the clearest priorities for strengthening that capacity.

## Supplementary material

10.2196/105709 Multimedia Appendix 1 Per-country values underlying the readiness analysis (research question 4).

## Abbreviations

CAGR: compound annual growth rate
GCC: Gulf Cooperation Council
GDHM: Global Digital Health Monitor
HIRI: Health Informatics Readiness Index
ITU: International Telecommunication Union
PRISMA: Preferred Reporting Items for Systematic Reviews and Meta-Analyses
STROBE: Strengthening the Reporting of Observational Studies in Epidemiology
UAE: United Arab Emirates
WHO: World Health Organization
