# Supplementary material for: Capacity, Collaboration, and Thematic Structure in Health AI Research Over the Past Decade in the Gulf Cooperation Council: Scientometric Analysis
Source: JMIR Med Inform. 2026 Oct 21;14:e105709. doi: 10.2196/105709 (PMC13686240; doi:10.2196/105709)
Supplement: Supplementary file 1 [file medinform-v14-e105709-s001.docx]

# **Multimedia Appendix 1**

*Per-country values underlying the readiness analysis (Research Question 4).*

This appendix reports the national values used in the exploratory correlation of health-AI research with informatics readiness and digital-health maturity for the six Gulf Cooperation Council (GCC) states, 2011–2024. Absolute output (Works) and per-capita intensity (publications per million population) are reproduced from Table 1 of the main text. The Health Informatics Readiness Index (HIRI) composite is the equal-weight national score; national health-AI intensity correlates with HIRI at Spearman ρ = +0.60 (n = 6). Absolute output tracks Global Digital Health Monitor (GDHM) maturity phase at Spearman ρ = +0.83 (n = 6).

**Table A1.** National health-AI output, intensity, HIRI composite, and GDHM phase.

| **Country** | **Works, n** | **Intensity (per million pop.)** | **HIRI composite (equal-weight)** | **GDHM phase** |
| --- | --- | --- | --- | --- |
| Qatar | 367 | 135.9 | 90.8 | [confirm] |
| United Arab Emirates | 768 | 80.8 | 88.3 | [confirm] |
| Saudi Arabia | 2,094 | 62.9 | 77.3 | [confirm] |
| Bahrain | 47 | 31.3 | 77.5 | [confirm] |
| Oman | 125 | 27.2 | 61.0 | [confirm] |
| Kuwait | 111 | 25.8 | 84.2 | [confirm] |

Abbreviations: GCC, Gulf Cooperation Council; HIRI, Health Informatics Readiness Index; GDHM, Global Digital Health Monitor; pop., population.

Notes. Works and intensity are from Table 1 of the main manuscript. HIRI composite scores are the equal-weight national values; the intensity–HIRI ranking reproduces Spearman ρ = +0.60 (n = 6). Countries are ordered by per-capita intensity (highest first). Correlations are descriptive; with six countries none reaches statistical significance, and Research Question 4 is reported as exploratory in the main text.

Author action required before submission: (1) enter each country’s GDHM maturity phase (ordinal 1–5) from the Global Digital Health Monitor source used, replacing the “[confirm]” placeholders, and verify that the Works–GDHM ranking reproduces Spearman ρ = +0.83; (2) confirm the exact HIRI composite scores against the HIRI source (reference 12), as the values shown here were read from Figure 6 and may differ from the source in the second decimal place.
